# The Prevalence and Awareness of Cardiovascular Diseases Risk Factors among the Lebanese Population: A Prospective Study Comparing Urban to Rural Populations

**DOI:** 10.1155/2017/3530902

**Published:** 2017-03-30

**Authors:** Iqbal Fahs, Zainab Khalife, Diana Malaeb, Mohammad Iskandarani, Pascale Salameh

**Affiliations:** ^1^Department of Pharmaceutical Sciences, School of Pharmacy, Lebanese International University, Beirut, Lebanon; ^2^Faculty of Pharmacy, Lebanese University, Hadath Campus, Beirut, Lebanon; ^3^Faculty of Medicine, Lebanese University, Hadath Campus, Beirut, Lebanon

## Abstract

*Introduction*. CVDs are largely driven by modifiable risk factors. This study sought to determine the awareness and prevalence of the modifiable CVDs risk factors among the Lebanese population.* Methods*. In a cross-sectional survey, 1000 participants aged ≥ 45 years were randomly selected from pharmacies and interviewed. The data was analyzed with SPSS version 21.0 software.* Results*. Differences between urban and rural areas include alcohol consumption (2.8% versus 1.7%; *p* = 0.0001), cardioprotective vegetable servings (6.1% versus 2.3%; *p* = 0.016), sedentary hours per day (18.6% versus 15.1%; *p* = 0.002), and hypertension (38.5% versus 25.4%; *p* = 0.001). The prevalence of overweight and obesity (77.3% versus 75.2%; *p* = 0.468), smoking (39.3% versus 43.3%; *p* = 0.232), diabetes (25.4% versus 21.4%; *p* = 0.173), and dyslipidemia (25 versus 21.2%) was reported. Measurements revealed 19.3% of undiagnosed hypertension (12.4% versus 22.4%, *p* = 0.001), 61.7% of hypertension (59.8% versus 62.6%; *p* = 0.203), and 7.9% of undiagnosed diabetes (6.6% versus 8.6%; *p* = 0.323). The declared awareness of CVDs risk factors was highest for smoking (91.5% versus 89.7%; *p* = 0.339) and lowest for diabetes (54.4 versus 55.7%; *p* = 0.692).* Conclusion*. This study has shown a high prevalence of modifiable CVDs risk factors in the Lebanese population ≥ 45 years, among which hypertension is the most prominent.

## 1. Introduction

Cardiovascular diseases (CVDs) are common in the general population, mainly among adults [1]. Based on different epidemiological studies, several nonmodifiable and modifiable CVDs risk factors have been identified. Advanced age, family history, and male gender are nonmodifiable risk factors [2]. Modifiable risk factors include smoking, hypertension, diabetes, dyslipidemia, obesity, physical inactivity, unhealthy diets, and heavy alcohol intake [2].

Cardiovascular diseases are the leading cause for death accounting for more than 17 million deaths each year (30% of all deaths) [1]. In the Middle East, the rate of increase in CVD-associated mortalities is one of the highest rates in the world [1]. In 2010, ischemic heart disease was the leading cause of death in Lebanon (32.2%), according to a study on the Burden of Disease and Risk Factor [3]. Several international [4–8], nearby [9–13], and national [14–16] studies had investigated the prevalence of CVDs risk factors. Based on national studies [17–19] the prevalence rates of CVDs risk factors are following an alarming trend over time. Individuals' knowledge of CVDs risk factors is prominent in targeting these factors. However, few studies worldwide had addressed the knowledge of CVDs risk factors [20–22].

There is an emerging increase in the prevalence of CVDs risk factors in the Middle East. Lebanese population ≥ 45 years of age bears a substantial burden of modifiable CVDs risk factors. In a presidential advisory issued in 2012, the presidents of leading cardiac societies called to reduce deaths from noncommunicable by 25% by the year 2025 [23]. Based on this recommendation and since the Lebanese awareness of CVDs risk factors has not been yet assessed, this study was conducted. The current study was designed to investigate the prevalence and awareness of CVDs risk factors among Lebanese individuals 45 years of age and above in urban and rural areas.

## 2. Methods

### 2.1. Study Design

This is a cross-sectional survey conducted all over Lebanon. The sample was drawn randomly from community pharmacies based on stratified cluster sampling. The strata were the districts of Lebanon that includes the capital Beirut and five other districts (Mount Lebanon, North, Beqaa, Nabatiye, and the South) that are mixture of rural villages and urban cities. Clusters were further selected at the level of districts (urban and rural areas). A total of 96 community pharmacies constituted the primary sampling units. A list of all community pharmacies in Lebanon was obtained from the Order of Pharmacists in Lebanon (OPL) website; the list was further divided into two lists of urban and rural community pharmacies per each district. Using Research Randomizer computer program, 8 community pharmacies were randomly selected from each list. 1000 individuals entering these pharmacies to purchase medications or parapharmaceuticals were actively recruited in March 2015, out of 1253 approached individuals and with a refusal rate of 20.19%. During the interview, patient education about CVDs risk factors was done directly by the interviewing pharmacists. Since CVDs morbidity and mortality increase with age, all individuals ≥ 45 years of age were considered eligible for study enrollment. Pregnant and lactating women, cancer patients, individuals with mental illnesses, and patients with established CVDs were excluded from the study. The study was approved by the ethical committee at the Lebanese International University.

### 2.2. Data Collection Sheet

The data collection sheet (see S1 Appendix in Supplementary Material available online at https://doi.org/10.1155/2017/3530902) used during interviews was established based on validated and standardized questionnaires that included WHO STEPS instrument guideline for noncommunicable diseases [24], Behavioral Risk Factor Surveillance System (BRFSS) [25], International Physical Activity Questionnaire (IPAQ) [26], Food Frequency Questionnaire (FFQ) [27], Adult Questionnaire [28], and Heart Disease Fact Questionnaire [29]. Written informed consent was obtained initially from subjects after thorough explanation. Data were stripped of any personal identifiable information.

### 2.3. Measurements

For the assessment of obesity, the height and weight were measured by standardized techniques, based on the recommendations of NHANES (National Health and Nutrition Examination Surveys) anthropometry and physical activity monitor procedures manual [30]. Individuals were weighed bare feet using an electronic scale (LAICA®, Personal Scale; Mod. PS50090) in light indoor clothing and recorded to the nearest 0.1 Kg. Height was measured without shoes using portable stadiometer (Seca®, 213) and recorded to the nearest 0.5 cm. Based on JNC 7 [31] recommendations for blood pressure measurement, systolic and diastolic blood pressures were measured for three readings with a digital blood pressure (Omron®, M3 IT) machine, each separated by 5 min of rest in between. The participants were asked to sit in a chair with the back supported and the arm at the level of the heart. Tobacco, alcohol, and caffeine were not allowed for at least 30 minutes before taking the measurements. An average of the second and the third reading was taken. Random blood glucose measurement was taken with a digital glucose meter (Accu-Chek® Performa) following the procedure explained in the manual [32]. Lipid panel measurements were retrieved from the participants, if available.

CVDs risk factors awareness is the individual's knowledge of the diseases, nutritional habits, or lifestyle behaviors that can result in a cardiovascular disease if left untreated or modified. The awareness of CVDs risk factors among our participants was assessed based on eight questions from Heart and Disease Fact Questionnaire [29] retrieving knowledge regarding smoking, obesity, alcohol consumption, improper diet, physical inactivity, hypertension, diabetes, and dyslipidemia. The awareness was computed as a continuous variable.

Atherosclerotic CVD (ASCVD) risk was calculated using Pooled Cohort Equations for patients with available lipid panel record and age less than or equal to 79 years [33]. ASCVD risk score is based on age, gender, SBP and treatment status, total cholesterol level, and HDL level [33]. The projected 10-year risk calculated was further classified into low risk (≤10%), intermediate risk (10–20%), and high risk (≥20%).

### 2.4. Study Outcomes

The main primary outcome measures the prevalence of modifiable CVDs risk factors and awareness among Lebanese population in urban and rural areas.

### 2.5. Sample Size Calculation and Statistical Analysis

The sample size (*n* = 1000) was calculated using an alpha of 0.05 and a power of 80%. Data analysis was conducted with the statistical software package “Statistical Package for Social Sciences” software (SPSS, version 21). Descriptive statistics were used to describe patient characteristics and mean for continuous variables. The studied groups were compared for statistically significant differences using chi-square test for nominal variables and analysis of variance (ANOVA) test for continuous variables. Linear regression was used to test for significant correlations between nominal or continuous variables and continuous outcomes, while binary logistic regression was used to test for significant associations between nominal or continuous variables and nominal outcomes. All reported *p* values were two-sided with the alpha set at a significance of 0.05.

## 3. Results

### 3.1. Baseline Characteristics

At baseline, 1000 participants aged ≥45 years were approached in different urban (*N* = 333, 33.3%) and rural (*N* = 667, 66.7%) areas across Lebanon. The study population included 501 men (50.1%) and 499 women (49.9%) with a mean age of 54 ± 15.12 years. The distribution and characteristics of demographic variables among the two studied groups (urban and rural) are summarized in Table 1.

The average body mass index (BMI) of the study population was 28.20 ± 4.50. Almost half of the sample (50.1%) had healthcare coverage. Urban participants were more educated (34.9% versus 19.8%, *p* = 0.0001), more able to seek medical care (72.6% versus 65.1%, *p* = 0.017), and less employed (28.6% versus 44.4%, *p* = 0.0001) than their rural counterparts. Urban participants received higher incomes (high income: 22.6% versus 13.3%, *p* = 0.0001) and had higher prevalence of family history of hypertension (65.5% versus 54.1%, *p* = 0.001).

### 3.2. Prevalence of Modifiable CVDs Risk Factors

Data of the traditional modifiable CVDs risk factors are presented in Table 2. Among the parameters studied, the most prevalent risk factor was overweight and obesity and the least prevalent was heavy alcohol intake. The prevalence of these risk factors did not differ significantly between the two studied groups (urban and rural) except for alcohol consumption, vegetable servings, exercise, and hypertension. 42% (401) of the studied population reported smoking either cigarettes or waterpipe. Only 2.1% (20) reported heavy alcohol consumption of ≥3 drinks/day. The prevalence of alcohol intake significantly differed between the two studied groups with more rural inhabitants being nonalcoholic (87.5% versus 72.9%, *p* = 0.0001).

Only 17.3% (172) and 8.7% (86) reported not consuming at all fruits and vegetables, respectively. However, a low proportion of the participants 8.1% (81) and 3.5% (35) consumed cardioprotective servings/day of ≥4 for fruits and ≥5 for vegetable, respectively.

The prevalence of moderate exercise and walking was high, where 54.5% (539) and 70.0% (696) reported engaging in moderate activities and walking for at least 10 minutes for ≥4 times/week. Urban inhabitants spent more sedentary sitting hours of ≥7 hours/day (62.8% versus 50.9%, *p* = 0.002). 46.5% (461) of the study participants were overweight and 29.4% (291) were obese with no significant difference in terms of residence (*p* = 0.468).

The prevalence of reported hypertension, diabetes, and dyslipidemia previously diagnosed by a doctor was 29.8% (294), 22.8% (215), and 22.5% (183), respectively. There was no significant between the two studied groups except for hypertension (*p* = 0.001), where the prevalence of reported hypertension was higher among urban participants (38.5% versus 25.4%, *p* = 0.001).

### 3.3. Measured Blood Pressure

**Figure 1:**
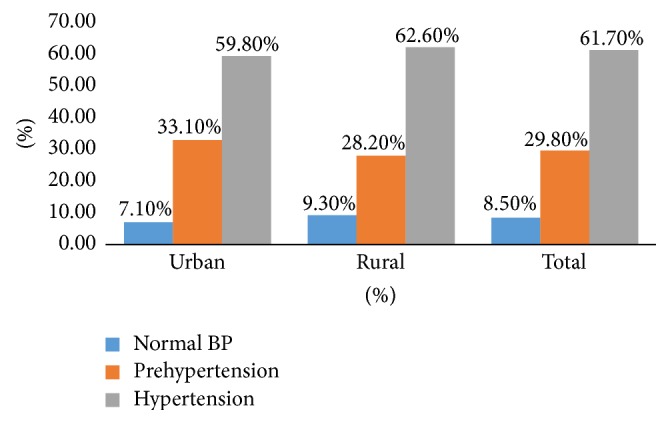
Prevalence of normal BP, prehypertension, and hypertension.

The average systolic blood pressure (SBP) and diastolic blood pressure (DBP) were 131.54 ± 16.07 and 80.85 ± 9.81 (Table 3), respectively. The blood pressure (BP) measurements were further classified into 3 groups (Figure 1): normal BP (SBP < 120 and/or DBP < 80 mmHg), prehypertension (SBP 120–139 and/or DBP 80–89 mmHg), and hypertension (SBP ≥ 140 years and/or DBP ≥ 90 mmHg).

Almost two-third of the participants 61.7% (59.8% versus 62.6%; *p* = 0.2030) were hypertensive, 29.8% (33.1% versus 28.2%; *p* = 0.203) were prehypertensive, and only 8.5% (7.1% versus 9.3%; *p* = 0.203) had normal BP measurements. No significant differences in measured blood pressure were revealed between the two groups except in undiagnosed hypertension. Based on BP measurements among those who were previously healthy, the prevalence of undiagnosed hypertension was 19.3% (160) with a higher prevalence among rural participants (12.4% versus 22.4%, *p* = 0.001). Undiagnosed hypertension significantly correlated with different variables (Table 4). Those who were counseled by their pharmacists during each visit and those who had higher educational and income levels had lower prevalence of undiagnosed hypertension.

### 3.4. Blood Glucose Measurement

The random blood glucose (RBG) measurement of the studied population ranged between 60 mg/dL and 359 mg/dL with an average of 123.03 ± 52.68 and no statistical significant difference in terms of residence (*p* = 0.153, Table 3). The prevalence of undiagnosed diabetes defined as RBG ≥ 200 mg/dL was 7.9% (6.6% versus 8.6%; *p* = 0.323) with no significant difference between the two studied groups. Undiagnosed diabetes significantly correlated with pharmacist counseling to participants during each visit (*p* = 0.0001, Table 4).

### 3.5. Lipid Profile

The baseline averages of triglycerides, total cholesterol, high-density lipoprotein, and low-density lipoprotein were 195.26 ± 85.08, 217.01 ± 47.99, 42.87 ± 10.63, and 137.51 ± 49.23, respectively. The lipid profile was not significantly different between urban and rural areas (Table 3).

### 3.6. CVDs Risk Factors

The average of CVDs risk factors was 2.06 ± 1.02 (Table 3) with no significant differences between the two studied groups (*p* = 0.107). Those who were older, males, had lower educational level and higher BMI had a higher average of CVDs risk factors (Table 4).

### 3.7. CVDs Risk Factors Awareness

**Figure 2:**
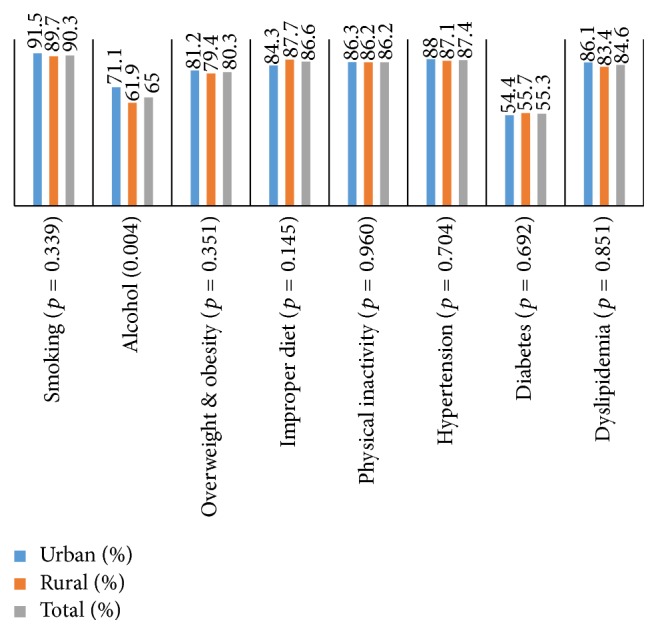
Awareness of modifiable CVDs risk factors among the study participants.

The study participants were mostly aware of smoking as a CVD risk factor and at least aware of diabetes (Figure 2). The mean awareness score was 5.67 ± 1.41 (Table 3) with no significant differences between the two studied groups (*p* = 0.77). The awareness score significantly correlated with different variables (Table 4). Those who were counseled by their pharmacists, had healthcare coverage, were able to seek medical care, were more educated, were employed, had higher income levels, have family history of diabetes, walked more, sat less, and had lower BP and BG measurements had higher awareness score.

### 3.8. ASCVD Risk

**Figure 3:**
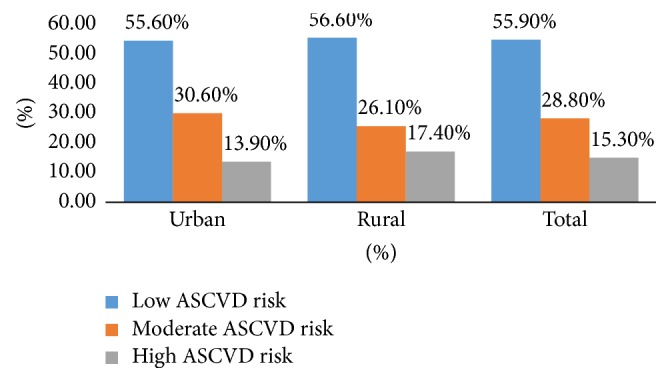
ASCVD risk in the study population.

The mean of ASCVD risk was 11.00 ± 8.38 (Table 3) with no significant difference between urban and rural participants. ASCVD significantly correlated with different variables (Table 3). Females, nonsmokers, and those who were counseled by pharmacists, sat less, and had lower BG measurement had lower ASCVD risk. ASCVD risk (Figure 3) was further categorized into 3 groups, low risk (<10%), moderate risk (10–20%), and high risk (>20%).

### 3.9. Counseling and Monitoring Parameters

The counseling and monitoring parameters data at baseline are summarized in Table 5. Urban participants were more counseled by pharmacist during each visit (52.0% versus 42.3%, *p* = 0.004). They self-monitored their blood pressure more frequently (23.7% versus 15.9%, *p* = 0.004). Blood glucose level was more monitored in urban areas with at least once/year measurement by 43.75 versus 31.3% in rural areas (*p* = 0.004). 50% (144) of urban participants monitored their lipid profile every 1-2 years versus only 38.9% (202) among rural participants (*p* = 0.014) (Table 5).

## 4. Discussion

In this study, urban participants were more educated, more able to seek medical care, and more counseled by their pharmacists and monitored their BP, BG, and lipid profile more frequently. This is mostly due to higher income levels attained in urban settings, easier healthcare access, and better transportation and teaching facilities.

The second most prevalent CVDs risk factor among our studied population was smoking (42%), which is comparable to the results of the Initiative for Cardiovascular Service in the Primary Healthcare done in 2013 in Lebanon [16]. There was no significant difference in smoking prevalence between urban and rural areas. On the contrary to our study, the results of Idris et al.'s study [34] done in six Western European countries (Sweden, Finland, Denmark, Germany, Italy, and Spain) revealed a higher smoking prevalence in urban areas. Such results in our study can be explained by the increased rate of urbanization in Lebanon.

Although this study demonstrated quite high intake of fruits and vegetables among the participants, only a small proportion was consuming cardioprotective serving ≥5 of vegetables (3.5) and ≥4 for fruits (8.1%). These results were similar to those reported in Al-Nooh et al.'s study [9] done in the Kingdom of Bahrain where only 4.9% consumed ≥5 servings/day of fruits and vegetables. While comparable intake between urban and rural areas was reported in Sachdeva et al.'s study [35], a lower vegetable intake of cardioprotective servings among rural participants was noticed in our study. This can be due to better knowledge and awareness of the cardioprotective effects of vegetables among urban counterparts.

Moreover, 23.8% and 79.7% reported at least 10 minutes of heavy and moderate physical exercise, respectively. These figures are higher than those reported previously in Lebanon in Sibai et al. [15], where 18% and 42% reported engagement in heavy and moderate physical activities, respectively. Such better physical activity profile in our study might be due to our older population where physical activity is always encouraged to avoid and control chronic diseases. Moreover, the rural participants were more engaged in moderate activities and spent less sedentary sitting hours compared to urban participants. This is mostly associated with the type of employment and outdoor physical activities in each area. Unlike rural participants, urban individuals have few opportunities to engage in outdoor physical activities with a higher rate of office employment.

The overall prevalence of overweight and obesity in our study was 75.9% with 46.5% being overweight and 29.4% being obese. Similar results were obtained in 2013 in Yamout et al.'s study [16] in Lebanon, where 40% were overweight and 30.3% were obese. The overall prevalence of overweight and obesity was also comparable to that of Al-Nooh et al.'s study [9] in Bahrain, where the figure was 78.4%. Comparable to our results where no significant difference was revealed between the two groups, a study in United states (Befort et al. [36]) demonstrated that the rural-urban obesity disparity existing in younger Americans, aged 20–39, does not exist in older age groups. It is mostly probable that the dietary patterns and the sedentary lifestyles have contributed to such alarming rates of overweight and obesity.

The prevalence of reported hypertension (29.8%) was higher than that reported in Lebanon in Yamout et al.'s study [16] (13.3%), but lower than that demonstrated in Bahrain in Al-Nooh et al.'s study [9] and in Nigeria in Oguoma et al.'s study [6], where the prevalence was 36.9% and 35.7%, respectively. Hypertension was more reported in urban areas than rural ones (38.5% versus 25.4%). Similarly, an Indian study (Bhansali et al. [37]) reported significantly higher hypertension prevalence among urban participants. This is mainly due to the behavioral lifestyles and better healthcare access at urban areas. Based on real measurements, the overall prevalence of hypertension and prehypertension was 61.7% and 29.8%, respectively. Those alarming rates might be due to the older population enrolled, low rates of regular medical checkups, and high rates of stress and to the fact that most women in this age group are past menopause.

The prevalence of reported diabetes (22.8%) was higher than that reported previously in Lebanon in Sibai et al.'s study [15] after stratifying for age ≥ 50 years (18%) and much higher than that reported in Nigeriain Oguoma et al.'s study [6] (5.4%). In contrast to our study Mohan et al.'s study [38] had reported a higher prevalence of diabetes among urban individuals.

Our study has revealed a relatively good knowledge of CVDs risk factors. Similarly, in Kuwait in Awad and Al-Nafisi study [21] smoking was identified as a CVDs risk factor by over four-fifths of the participants as well. The mean of ASCVD risk (10 years to develop a CVD) in our study was 11.00 ± 8.38 with 55.9%, 28.8%, and 15.3% having low, moderate, and high risk, respectively. The percentage of high ASCVD risk was lower than that demonstrated in Baynouna et al.'s study [12] in United Arab Emirates (Baynouna et al., 2008) where 28.4% had a Framingham risk assessment score of >20%. The average number of CVDs risk factors and ASCVD risk were comparable in urban and rural areas, which could be explained by the increased urbanization rate in Lebanon.

This study has several limitations; the study lacks a national comparator group for the CVDs risk factors awareness, ASCVD risk, number of risk factors, and the monitoring parameters. Despite the presence of nearby and international studies, these may not reflect comparable backgrounds. Second, due to the absence of financial support, lipid profiles (94 profiles) reported by the participants were used. In addition, we were unable to measure FBG because the participants were approached throughout the whole day. That constituted a barrier to determine the prevalence of prediabetes due to the lack of a cut point in guidelines to estimate prediabetes based on RBG measurements. Being an observational study, there was no control over the participants' characteristics with possible sources of bias such as sampling, measurements, and information. Despite these limitations this is the first study in Lebanon that assesses the awareness of CVDs risk factors. It provides an accurate estimate of the prevalence of CVDs risk factors in a large adult population, in which intervention is possible.

## 5. Conclusion

Despite profound awareness about most CVDs risk factors, this study has shown significant prevalence of modifiable CVDs risk factors in urban and rural areas of Lebanon. This reflects alarming public health concerns and future demands that require instant interventions.

## Supplementary Material

The supplementary material S1 Appendix is the data collection sheet that was established based on validated questionnaires. This sheet was used during interviews with the participants to collect data required for the study.

## Figures and Tables

**Table 1:** Baseline characteristics of the study participants.

	Urban	Rural	Total	*p* value
	% (*N*)	% (*N*)	% (*N*)	
	33.3 (333)	66.7 (667)	100 (1000)	
Age (M ± SD)	54.41 ± 8.47	55.05 ± 10.06	54.84 ± 15.12	**0.321**
BMI (M ± SD)	28.22 ± 4.42	28.19 ± 4.55	28.20 ± 4.50	**0.926**
BMI categorized				**0.643**
Underweight	0.6 (2)	0.8 (5)	0.7 (7)	
Normal	22.1 (73)	24.1 (159)	23.4 (232)	
Overweight	49.4 (163)	45.1 (298)	46.5 (461)	
Obese	27.9 (92)	30.1 (199)	29.4 (291)	
Gender				**0.032**
Male	55 (183)	47.7 (318)	50.1 (501)	
Female	45 (150)	52.3 (349)	49.9 (499)	
Educational level				**0.0001**
Illiterate	9.3 (31)	12.3 (82)	11.3 (113)	
School	55.7 (185)	67.8 (453)	63.9 (638)	
University	34.9 (116)	19.8 (132)	24.8 (248)	
Marital status				**0.015**
Single	8.4 (28)	7.8 (52)	8 (80)	
Married	77.7 (258)	84.3 (562)	82.1 (820)	
Divorced	9.3 (31)	4.5 (30)	6.1 (61)	
Widow	4.5 (15)	3.4 (23)	3.8 (38)	
Occupational status				**0.0001**
Employed	28.6 (95)	44.4 (295)	39.2 (390)	
Average income per month				**0.0001**
Low	19.6 (65)	27.8 (184)	25.0 (249)	
Medium	57.8 (192)	59 (391)	58.6 (583)	
High	22.6 (75)	13.3 (88)	16.4 (163)	
Heath care coverage	51.5 (171)	49.8 (330)	50.4 (501)	**0.606**
Unable to seek medical care	27.4 (91)	34.9 (230)	32.4 (321)	**0.017**
Family history of CVDs	44.8 (147)	41.8 (278)	42.8 (425)	**0.367**
Family history of hypertension	65.5 (216)	54.1 (357)	57.9 (573)	**0.001**
Family history of diabetes	61.2 (202)	56.6 (376)	58.1 (578)	**0.168**
Family history of dyslipidemia	45.9 (151)	44.4 (295)	44.9 (446)	**0.661**

M: mean; *N*: number; SD: standard deviation.

**Table 2:** Prevalence of modifiable cardiovascular risk factors among the study participants.

Risk factor	Urban	Rural	Total	*p* value
	% (*N*)	% (*N*)	% (*N*)	
Smoking				**0.232**
Current smoker	39.3 (127)	43.3 (274)	42 (401)	
Passive smoker	10.5 (34)	8.9 (56)	9.4 (90)	
Ex-smoker	10.8 (35)	7.4 (47)	8.6 (82)	
Never smoked	39.3 (127)	40.3 (255)	40 (382)	
Alcohol (*N* of drinks)				**0.0001**
0	72.9 (237)	87.5 (568)	82.6 (805)	
1	15.4 (50)	7.7 (50)	10.3 (100)	
2	8.9 (29)	3.1 (20)	5 (49)	
≥3	2.8 (9)	1.7 (11)	2.1 (20)	
Diet				
Fruit servings/day				**0.122**
None	19.8 (65)	16.1 (107)	17.3 (172)	
1–3	70.5 (232)	76.5 (509)	74.5 (741)	
≥4	9.7 (32)	7.4 (49)	8.1 (81)	
Vegetables servings/day				**0.016**
None	9.4 (31)	8.3 (55)	8.7 (86)	
1-2	57.1 (188)	63.4 (421)	61.3 (609)	
3-4	9.1 (90)	17.4 (173)	26.5 (263)	
≥5	6.1 (20)	2.3 (15)	3.5 (35)	
Exercise at least 10 min				
Vigorous/week				**0.062**
None	75.6 (248)	76.5 (507)	76.2 (755)	
1–3	19.8 (65)	15.7 (104)	17.1 (169)	
4–6	1.8 (6)	4.5 (30)	3.6 (36)	
≥7	2.7 (9)	3.3 (22)	3.1 (31)	
Moderate/week				**0.014**
None	20.9 (68)	20.1 (133)	20.3 (201)	
1–3	29.4 (96)	23.1 (153)	25.2 (249)	
4–6	20.2 (66)	17.5 (116)	18.4 (182)	
≥7	29.4 (96)	39.4 (261)	36.1 (357)	
Walk/week				**0.001**
None	12.2 (40)	15.9 (106)	14.7 (146)	
1–3	10.4 (34)	17.7 (118)	15.3 (152)	
4–6	12.2 (40)	14.1 (94)	13.5 (134)	
≥7	65.2 (214)	52.3 (348)	56.5 (562)	
Sitting hours/day				**0.002**
1–6	37.2 (122)	49.1 (325)	45.2 (447)	
7–11	44.2 (145)	35.8 (237)	38.6 (382)	
≥12	18.6 (61)	15.1 (100)	16.3 (161)	
Overweight and obesity	77.3 (255)	75.2 (497)	75.9 (752)	**0.468**
Reported hypertension	38.5 (126)	25.4 (168)	29.8 (294)	**0.001**
Reported diabetes	25.4 (80)	21.4 (135)	22.8 (215)	**0.173**
Reported dyslipidemia	25 (70)	21.2 (113)	22.5 (183)	**0.226**

Min: minute; *N*: number.

**Table 3:** Average of measured and biochemical parameters at baseline.

Parameter	Urban	Rural	Total	*p* value
	M ± SD (*N*)	M ± SD (*N*)	M ± SD (*N*)	
SBP (mmHg)	130.87 ± 15.69 (310)	131.87 ± 16.25 (627)	131.54 ± 16.07 (937)	**0.365**
DBP (mmHg)	80.44 ± 9.39 (310)	81.06 ± 10.01 (625)	80.85 ± 9.81 (935)	**0.367**
RBG (mg/dL)	126.64 ± 55.23 (285)	121.12 ± 51.22 (539)	123.03 ± 52.68 (824)	**0.153**
TG (mg/dL)	199.40 ± 105.33 (42)	189.46 ± 44.45 (30)	195.26 ± 85.08 (72)	**0.628**
TC (mg/dL)	214.69 ± 51.72 (42)	220 ± 43.24 (32)	217.01 ± 47.99 (74)	**0.632**
HDL (mg/dL)	40.92 ± 7.74 (40)	45.64 ± 13.42 (28)	42.87 ± 10.63 (68)	**0.071**
LDL (mg/dL)	136.90 ± 54.48 (40)	138.48 ± 40.63 (25)	137.514 ± 49.23 (65)	**0.901**
Awareness score	5.69 ± 1.42 (332)	5.66 ± 1.41 (664)	5.67 ± 1.41 (996)	**0.775**
ASCVD risk	11.09 ± 8.47 (36)	10.87 ± 8.43 (23)	11.00 ± 8.38 (59)	**0.925**
Average number of risk factors	2.12 ± 1.07 (333)	2.04 ± 0.99 (667)	2.06 ± 1.02 (1000)	**0.384**

BMI: body mass index; DBP: diastolic blood pressure; HDL: high-density lipoprotein; LDL: low-density lipoprotein; TC: total cholesterol; TG: triglycerides; RBG: random blood glucose; SBP: systolic blood pressure.

**Table 4:** Significant correlations.

Dependent variable (type)	Independent variable (type)	Test used	Standardized coefficient^*∗*^	*p* value
ASCVD risk (continuous)	BG measurement (continuous)	Linear regression	0.813	**0.0001**
	Gender (nominal)	Linear regression	0.457	**0.02**
	Pharmacist counseling (nominal)	Linear regression	0.282	**0.033**
	Smoking status (nominal)	Linear regression	0.782	**0.0001**
	Sitting hours/day (ordinal)	Linear regression	0.429	**0.011**

Awareness score (continuous)	Average income (nominal)	Linear regression	0.068	**0.005**
	Average SBP (continuous)	Linear regression	0.155	**0.001**
	Average DBP (continuous)	Linear regression	0.096	**0.024**
	Ability to seek medical care (nominal)	Linear regression	0.144	**0.0001**
	BG measurement (continuous)	Linear regression	0.590	**0.012**
	BP measured by healthcare provider (nominal)	Linear regression	0.112	**0.004**
	Educational level (nominal)	Linear regression	0.268	**0.000**
	Family history of diabetes (nominal)	Linear regression	0.086	**0.011**
	Healthcare coverage (nominal)	Linear regression	0.740	**0.018**
	Occupational status (nominal)	Linear regression	0.083	**0.023**
	Pharmacist counseling (nominal)	Linear regression	0.162	**0.0001**
	Sitting hours/day (ordinal)	Linear regression	0.172	**0.0001**
	Walking/week (ordinal)	Linear regression	0.171	**0.0001**

Average CVDs risk factors (continuous)	Age (continuous)	Linear regression	0.158	**0.0001**
	BMI (continuous)	Linear regression	0.616	**0.015**
	Educational level (nominal)	Linear regression	0.212	**0.0001**
	Gender (nominal)	Linear regression	0.225	**0.0001**

Undiagnosed hypertension (nominal)	Average income (nominal)	Logistic regression	1.145	**0.0001**
	Educational level (nominal)	Logistic regression	1.610	**0.001**
	Pharmacist counseling (nominal)	Logistic regression	0.808	**0.046**

Undiagnosed diabetes (nominal)	Pharmacist counseling (nominal)	Logistic regression	0.493	**0.0001**

^*∗*^ *β* for linear regression and Exp(*β*) for logistic regression.

BG: blood glucose; BP: blood pressure; BMI: body mass index.

**Table 5:** Counseling and monitoring parameters at baseline.

Parameter	Urban	Rural	Total	*p* value
	% (*N*)	% (*N*)	% (*N*)	
Pharmacist counseling	52.0 (171)	42.3 (281)	45.5 (452)	**0.004**
Blood pressure monitoring by a healthcare provider				**0.0001**
Never	10.7 (35)	15.6 (103)	14.0 (138)	
With every visit to health	57.3 (188)	39.8 (262)	45.6 (450)	
Care provider				
At least once/year	17.4 (57)	25.9 (171)	23.1 (228)	
At least once/2 years	14.6 (48)	18.7 (123)	17.3 (171)	
Self-monitoring of BP	23.7 (76)	15.9 (100)	18.5 (176)	**0.004**
BG monitoring				**0.004**
None	9.3 (25)	8.8 (45)	9.0 (70)	
Once/3 years	20.5 (55)	28.3 (145)	25.6 (200)	
Once/year	43.7 (117)	31.3 (160)	35.5 (277)	
>1/year	26.5 (71)	31.6 (162)	29.9 (233)	
Lipid profile monitoring				**0.014**
None	24.3 (70)	30.6 (159)	28.4 (229)	
Every 4–6 months	9.0 (26)	8.5 (44)	8.7 (70)	
Every 1-2 years	50.0 (144)	38.9 (202)	42.9 (346)	
Every 5 years	16.7 (48)	22.0 (114)	20.1 (162)	

BG: blood glucose; BP: blood pressure; *N*: number.
