# Supplementary material for: The Prevalence and Awareness of Cardiovascular Diseases Risk Factors among the Lebanese Population: A Prospective Study Comparing Urban to Rural Populations
Source: Cardiol Res Pract. 2017 Mar 30;2017:3530902. doi: 10.1155/2017/3530902 (PMC5390633; doi:10.1155/2017/3530902)
Supplement: Supplementary file 1 — The supplementary material S1 Appendix is the data collection sheet that was established based on validated questionnaires. This sheet was used during interviews with the participants to collect data required for the study. [file 3530902.f1.docx]

Supplementary Appendix (S1 Appendix)

CARDIOVASCULAR RISK ASSESSMENT

QUESTIONNAIRE

IQBAL FAHS, PharmD Candidate

ZAINAB KHALIFE, PharmD Candidate

**A. Patient`s Demographics:**

Patient`s Number: ……… Urban: Rural:

Name: ……………………………………………………. Phone Number: …………………………….

Age: ………………………. Gender: Male: Female:

Weight: …………….. Height: ……………. BMI: ………… IBW:…….

Allergy(s): ………………………………………………….

**B .Health Care Access:**

1-When was the last date a health care provider seen?

□Within the past year

□Within the past 2 years

□Within the past 5 years

□≥ 5 years ago

□Never

2-Are you counseled by your pharmacist at each pharmacy visit?

□Yes □No

3-Do you have any kind of health care coverage?

□Yes □No

4-Was there a time in the past 12 months when you needed to see a doctor but could not

Because of any of these: Cost, traveling, transportation, or family issues?

□Yes □No

**C. Socio-economical status:**

1-What is your educational level?

□Illiterate

□School level

□University level

2-What is your marital status?

□Single

□Married

□Divorced

□Widow

3-What is your occupational status?

□Does not work

□Works

4-Are you a health care provider?

□Yes □ No

5-What is your average income per month?

□ Low

□ Medium

□ High

**D. Patient awareness of contributing risk factors:**

1. Do you think that smoking may increase your risk of cardiovascular diseases?

□Yes □No

2. Do you think that alcohol consumption can increase the risk of cardiovascular diseases?

□Yes □No

3 .Do you think that improper diet can increase risk of CV diseases?

□Yes □No

4-Do you think that physical inactivity can increase the risk of developing

cardiovascular diseases?

□Yes □No

5-Do you think that hypertension can increase the risk of developing

cardiovascular diseases?

□Yes □No

6-Do you think that diabetes can increase the risk of developing cardiovascular

diseases?

□Yes □No

7-Do you think that dyslipidemia can increase the risk of developing cardiovascular

diseases?

□Yes □No

**E. Family History:**

1-Do you have a family history of cardiovascular diseases?

□Yes □No

2-Do you have family history of hypertension?

□Yes □No

3-Do you have family history of diabetes?

□Yes □No

4-Do you have family history of dyslipidemia?

□Yes □No

**F.Risk Factors Assessment:**

**I. Cardiovascular Disease:**

1-Had you ever had a heart attack or chest pain from heart disease (angina) or stroke?

□Yes □No

2-Are you currently taking any antiplatelet to prevent a heart disease?

□Yes □No

3- Which antiplatelet are you taking?

□Aspirin

□Clopidogrel

Other:…..

**II. Tobacco Use:**

1-Describe your current smoking status?

□ Current smoker

□ Passive smoker

□ Ex- smoker

□ Never smoked

2- If you are a current smoker,how many cigarettes and/or shisha sessions you have per day?

□ <20 cigarettes/day

□>20 cigarettes/day

□<1 shisha session/day

□>1 shisha session/day

3-Have you ever tried to stop smoking?

□Yes □No

**III. Alcohol Consumption:**

1-What is your average daily alcohol consumption?

□ 0 drinks

□ 1 drink

□ 2 drinks

□ ≥3 drinks

**IV. Diet:**

1-How often do you usually eat fried foods?

□Less than once a week

□1-2 times a week

□3-6 times a week

□Every day

2-How many servings of sweet foods like cakes, biscuits, candies or chocolate do

you consume a day?

□ None

□1-2 serves

□More than 2 serves

3- How many pieces of fruit do you usually eat per day?

□ None

□1-3 pieces

□≥4 pieces

4-How many serves of vegetables do you usually eat per day?

□ None

□1-2 serves

□3-4 serves

□≥5 serves

5- Do you often add salt to your food before or while eating it?

□Yes □No

6- Do you consume your meals based upon a fixed time?

□Yes □No

**V. Physical Activity:**

1- How many times do you have vigorous exercise per week for at least 10 mins ?

□None

□1-3 times

□4-6 times

□7 times

2- During the last week, how many times did you do moderate physical activities like carrying light loads, bicycling at a regular pace for at least 10 minutes?

□None

□1-3 times

□4-6 times

□7 times

3-During the last week, how many times did you walk for at least 10 minutes?

□None

□1-3 times

□4-6 times

□7 times

4-How many hours you are approximately used to sit a day?

□1-6 hours

□7-11 hours

□≥12 hours

**VI. Hypertension:**

1-How often do you have your blood pressure measured by a health care provider?

□ At least once every year

□ At least once every 2 years

□ With every visit to healthcare provider

□ Never

2-Do you have hypertension diagnosed by a doctor?

□Yes □No

3-Do you take any medications to treat hypertension?

□Yes □No

4-Do you self-measure your blood pressure at home?

□Yes □No

5-Measurment of blood pressure and HR:

Reading 1 ………….. Reading 2 ………… Reading 3 ………….

HR1:…... HR2:…. HR3: ……..

**VII. Diabetes**

1-Have you ever had your FBG or HbA1c done before?

□Yes □No

2- What is the frequency of blood glucose measurement?

□ More than once per year

□ Once per year

□ Once every 3 years

3-Do you have diabetes diagnosed by a doctor?

□Yes □No

4-How often do you self-monitor your blood glucose?

□At least 4 times a day

□At least once per day

□None

5- Have you ever had measured your HbA1c?

□ Yes □ No

6-Your HbA1c was:

□<6.5 % □≥6.5 %

7-Do you take insulin or anti-hyperglycemic medications to treat your diabetes?

□Yes □No

8-Measurment of blood glucose:

Measurement ………

**VIII. Dsylipidemia**:

1-Have you had your lipid profile measured before?

□Yes □No

2- How often do you have a complete lipid panel done?

□ Every 5 years

□ Every 1-2 years

□ Every 4-6 months

□ None

3-Do you have dyslipidemia diagnosed by a doctor?

□Yes □No

4-Are you currently taking any medication to treat dyslipidemia?

□Yes □No

5-Lipid profile measurement:

Total cholesterol ………. TG………. HDL ………. LDL ……….

**Patient ASCVD risk:**

**THANK YOU**
